# Canadian Men’s Intimate Partner Relationship Break-Ups During COVID-19: Implications for Mental Health Promotion

**DOI:** 10.1177/10497323241307195

**Published:** 2025-01-22

**Authors:** John L. Oliffe, Nina Gao, Matthew Sha, Lannea Niebuhr, Raymond Chou, Jennifer Mootz, Sarah McKenzie

**Affiliations:** 1 School of Nursing, 8166 University of British Columbia, Vancouver, BC, Canada; 2 Department of Nursing, University of Melbourne, Melbourne, VIC, Australia; 3 Department of Psychiatry, New York State Psychiatric Institute, 5798 Colombia University, New York, NY, USA; 4 Department of Psychological Medicine, 2495 University of Otago, Dunedin, New Zealand

**Keywords:** men’s mental health promotion, intimate partner relationships, relationship break-ups, masculinity, gender relations

## Abstract

COVID-19 impacted many men’s intimate partner relationships, with distressed and disrupted partnerships consistently featured in commentaries with linkages to mental health challenges. The current study draws from interviews with 23 Canadian-based men, 19–50 years old, who experienced a break-up during COVID-19. Addressing the research question, “What are the connections between masculinities, men’s mental health, and intimate partner relationship break-ups during COVID-19?”, three thematic findings were derived: (1) Virtually Together and Growing Apart, (2) Mentally Trapped, and Failing Fast and Slow, and (3) Introspections and Moving On. *Virtually Together and Growing Apart* describes two contexts wherein men were either challenged by being physically apart from their partner or increasingly estranged while cohabitating with their partner during COVID-19. *Mentally Trapped, and Failing Fast and Slow* speaks to the stresses of being socially isolated and anxieties about the future with those tensions flowing to and from men’s relationships. Featured were fast-tracked endings in terms of many participants knowing early-on the partnership was over, amid drawn out finishes wherein men’s relationships gradually ended with the easement of COVID-19 restrictions. *Introspections and Moving On* varied in that many men were intent on processing and deconstructing all that happened in (and to end) their relationship. Men’s learnings were leveraged through accessing professional and peer supports to promote self-growth and purposefully build healthier intimate partnerships. The study findings affirm the need for gender-responsive mental health promotion programs to equip men with relationship skills, while also underscoring the necessity for services dedicated to addressing post-COVID-19 injuries.

## Background

The impacts of COVID-19 have drawn commentaries and empirical insights to detail an array of pandemic-related social health issues (Abrams & Szefler, 2020). Among these, intimate partner relationships have featured, wherein increases in distressed and/or disrupted partnerships have consistently been linked to the pandemic (Herbenick et al., 2022), with predictions that the full extent of the brunt will unfold as we continue to transition out of COVID-19 (Apriasari et al., 2021). Men’s mental health is intricately tied to their intimate partner relationships, and in the specific context of men’s break-ups, divorced men are 2.8 times more likely to die by suicide than married men, with separated men under 34 years old having 8.6 times the suicide risk compared to their married counterparts (Wilson et al., 2023). While cause-effect hypotheses abound regarding the connections between men’s mental health and intimate partner relationship break-ups in COVID-19, qualitative research findings about masculinities and men’s lived experiences of disrupted partnerships in the pandemic are conspicuously lacking. Addressing this knowledge gap, the current article provides a thematic analysis distilling connections between Canadian-based men’s masculinities, mental health, and intimate relationship break-ups during COVID-19, as a means to guiding gender-responsive health promotion programs and services (Shah, 2023).

### Masculinities, Men’s Mental Health, Intimate Partner Relationships, and COVID-19

To reduce the spread of COVID-19, a wide range of lockdown measures were established in Canada, wherein, at various stages of the pandemic, there were international and local travel bans, cancellation of events, closed public spaces, physical distancing, and restricted social gatherings. Research has highlighted the negative effects of COVID-19 restrictions on men’s mental health, a trend that was also observed during the SARS and MERS outbreaks (Park & Yu, 2022). The specific COVID-19 impacts on men’s mental health include heightened risk for depression and anxiety (Park & Yu, 2022; Simpson et al., 2022) and increased rates of male suicide (Sacco et al., 2023). Linking these risks and outcomes to social health issues in a survey of 434 Canadian-based men, a third (31.1%; *N* = 135) suggested their living situation during lockdown had a considerable or severe negative impact on their mental health, with just under half (42.2%; *N* = 183) disclosing that they had experienced suicidal ideation (Ogrodniczuk et al., 2021). Deeply entwined with many men’s COVID-19-related mental health challenges were their intimate partner relationships (Luetke et al., 2020). About two-fifths (37.7%; *N* = 94) of the 249 partnered men indicated that COVID-19 had a negative impact on their intimate partner relationships, with nearly a third (30.9%; *N* = 77) disclosing that they engaged in abuse (primarily verbal, 22.9%; *N* = 57) toward their intimate partner, while more than a quarter (27.3%; *N* = 68) reported having been abused by their intimate partner (Ogrodniczuk et al., 2021). These trends were also evident in large population health data with a 4% rise in intimate partner violence (IPV) 2019 through 2021 (Statistics Canada, 2022b) and 2%–6% escalation in domestic violence (DV) and IPV among all genders in Canada (Statistics Canada, 2022a, 2023b).

Contextualizing these negative outcomes, parenting, finances, and domestic chores challenged many couples, with 38% also reporting disputes about COVID-19 regulations and 20% regarding vaccinations (Statistics Canada, 2023a). Goodboy et al. (2021) reported a positive relationship between intimate partner interference and negative emotions including anger, sadness, and fear. Similarly, Pietromonaco and Overall (2021) indicated that external stressors adversely affected relationships with decreased responsiveness to partners and reduced self-regulation. Consistently reported also were connections between low relationship quality and increased risk for depressive symptoms (Charvat et al., 2023; Zoppolat et al., 2022). Samokhvalova et al. (2022) delineated the stressors of long-term forced separation (through physical distancing) in close relationships during COVID-19 as resulting in loneliness, longing, and sadness. Confirming men’s social health challenges as widespread, a scoping review lobbied long-term follow-up care post COVID-19 to prevent men’s mental health issues progressing to chronic diseases (Park & Yu, 2022).

In recent non-COVID-19 intimate partner relationship research, Connell’s (2005) masculinities framework has been used to explain the gendered dimensions of men’s mental health challenges in distressed and/or disrupted partnerships (Oliffe, Kelly, Gonzalez Montaner, Seidler, Kealy, et al., 2022; Oliffe, Kelly, Gonzalez Montaner, Seidler, Ogrodniczuk, et al., 2022). Herein, the plurality of men’s masculine performativity has featured diverse behaviors ranging from maladaptive practices including substance use and anger toward ex-partners to strength-based reflexive work with the goal of purposefully securing self-growth after the break-up (Oliffe, Kelly, Gonzalez Montaner, Kealy, et al., 2022; Oliffe, Kelly, Gonzalez Montaner, Seidler, Kealy, et al., 2022; Oliffe, Kelly, Gonzalez Montaner, Seidler, Ogrodniczuk, et al., 2022). Within these studies, participants’ diverse alignments to idealized masculine norms of self-reliance, strength, and stoicism have been used to explain men’s deficits (e.g., concealment of mental health challenges and reticence for help-seeking) and gains (e.g., engaging professional help to improve effective self-management, and working to build accountability and relationship skills). Illuminating men’s pandemic specific experiences, the current study answers the research question: *What are the connections between masculinities, men’s mental health, and their intimate partner relationship break-ups during COVID-19*?

## Methods

Drawing from interpretive descriptive methodologies (Thorne & Francis, 2017), individual semi-structured Zoom interviews were conducted, and Connell’s (2005) masculinities framework was used to interpret, theorize, and conceptually advance the analyses and findings. In line with the applied focus of interpretive description (Thorne, 2016), the application of the current study findings to men’s mental health promotion programs and services is discussed with a view to advancing gender-responsive interventions (Gough & Novikova, 2020).

### Data Collection

With university ethics approval (H20-01868), the project manager reached out to waitlisted participants who had expressed interest in participating in a previous study focused on men’s experiences of and perspectives about equitable intimate partner relationships (Oliffe et al., 2023). The men on the waitlist were screened for the following eligibility: Canadian-based, English-speaking, 19 years old or older, and having had a relationship break-up during COVID-19 (March 2020–June 2022). Eligible participants completed a Qualtrics-hosted e-consent, demographics, and survey questionnaires, ahead of taking part in an individual Zoom interview (Oliffe et al., 2021) with one of 7 researchers (2 women and 5 men based in Canada). The Patient Health Questionnaire (PHQ-9) (Kroenke et al., 2001) was completed by participants, and the aggregate depression scores and classifications are reported along with suicidal ideation results (please see Table 1). Comprising 23 cisgender, childless men, ranging in age from 19 to 50 years old (M = 26.68; SD = 7.06), most participants self-identified as heterosexual (*N* = 19; 83%), and just over half (*N* = 12; 52%) had re-partnered at the time of interview. Participant interviews were conducted February through June 2023, took 40–68 minutes (M = 53 minutes; SD = 7.03 minutes), were audio-visually recorded in Zoom, and were professionally transcribed and checked for accuracy. Written summaries were completed for each participant interview, and the men were assigned a numeric identifier and pseudonym by the researchers.

**Table 1.** Participant Demographics.

Demographics, *N =* 23	*N* [%]
Age	
19–29	17 [74]
30–39	4 [17]
40–49	1 [4]
50–59	1 [4]
Education	
Some college	2 [9]
Bachelor’s degree	14 [61]
Master’s degree	5 [22]
Other	2 [9]
Employment status	
Employed for wages and studying full-time	3 [13]
Employed for wages	9 [39]
Studying full-time	6 [26]
Looking for employment	3 [13]
Not employed and not looking for employment	1 [4]
Prefer not to say	1 [4]
Sexual orientation	
Heterosexual	19 [83]
Gay	2 [9]
Bisexual	2 [9]
Relationship status	
Single	11 [48]
Partnered	12 [52]
PHQ-9 scale results	
None or minimal depression	13 [57]
Mild depression	4 [17]
Moderate depression	1 [4]
Moderately severe depression	3 [12]
Severe depression	2 [9]
Suicidal ideation in the last 2 weeks	
Yes	3 [13]
No	20 [87]
Who do you talk to about your relationship? (Select all that apply)	
Partner/spouse	8 [35]
Friends	20 [87]
Family	14 [61]
Health care worker (doctors, therapists, counsellors, social workers)	4 [17]

### Data Analysis

Using constant comparison analytics, each transcript was read in full and compared to develop descriptive labels under which data segments were coded. Independently reading the interview transcripts, the research team met to discuss preliminary interpretations and plans for organizing the data with the goal of making broad fractures to retain the context of the interview while assigning and managing excerpts relevant to the research question. Initial descriptive codes included physically trapped, temporally suspended, fast-forward endings, and mental health. In assigning interview data to the codes, some descriptors were subsumed, and consensus was built through researcher meetings to assign data to four codes: temporally suspended, mentally trapped, accelerated endings, and lessons. Reviewing the data in each code, patterns and variations were inductively derived, and as the analyses progressed, descriptive labels were transitioned to theme headers to pre-empt the key findings. The analyses were further advanced by applying the masculinities framework and in the writing-up of the findings and this article, with reflexive memos written to guide and report the analytics used to develop the three themes: (1) Virtually Together and Growing Apart, (2) Mentally Trapped, and Failing Fast and Slow, and (3) Introspections and Moving On. While the first theme, *Virtually Together and Growing Apart*, differentiates participants who were living separately to their partner from men who were cohabitating with their partner during COVID-19, themes 2 and 3 report patterns across the 23 men.

## Results

### Virtually Together and Growing Apart

Participant narratives about their relationship break-ups varied based on their living arrangements. Those who lived separately from their partner (*N* = 17) described challenges related to being virtually together, while men who cohabitated (*N* = 6) with their partner spoke about growing apart while living together during the pandemic. The men who were physically separated from their partner discussed wide-ranging factors as contributing to their break-ups. Charlie, a 27-year-old heterosexual man, detailed his 3-year relationship—explaining that when the COVID-19 travel restrictions were put in place, he was living and studying in the United Kingdom, and his partner was in Hong Kong with the plan for meeting later in 2020 nixed by the pandemic. Speaking to the limits of connecting virtually, he explained:

Any long-distance Zoom call or video call—or any form of electronic communications … although it does help to maintain your relationship a little bit, it’s not actually quality time. We would go on streaming party on Netflix or whatever, try to watch something together. But the feeling is just not the same … COVID, it takes away all of that.

Contrasting the 2 years in which they had lived together prior to the pandemic, Charlie suggested that being stuck at home with “nothing going on in life” seeded (and cemented) their disconnection: “The video call duration started to drop because we basically didn’t have anything to talk to each other about.” Also implicated were Charlie’s omissions about his mental health challenges and self-medicating with alcohol, redactions he premised on wanting to “shield her [partner] from all this stress.” While Charlie reasoned this as protective of his partner (i.e., not adding to her pandemic-related distress), he struggled with being alone, lamenting, “being caged, feeling helpless, feeling unsupported, feeling lonely … your mind is just not reliable.” Evident here was Charlie’s alignment to masculine ideals for being self-reliant and emotionally stoic under the guise of protecting his partner. Somewhat ironically, these concealments further distanced Charlie from his partner, resulting in a vicious cycle of being stuck in his worsening mental health challenges and a distressed partnership.

The centrality of the pandemic in the everyday also subdued affections and emotional intimacy when men virtually connected with their partners. Owen, a 24-year-old heterosexual man whose 2.5-year relationship switched from in-person to long-distance just prior to the pandemic, explained the void of being in separate worlds:

I’m more of an emotional person. So, like, physicality is a big thing for me. And I like to read body language and see how someone’s feeling and everything like that. And obviously, through text messages … it’s pretty much impossible, right. You can speak to someone on the phone … but again … being in physical presence with someone is completely different.

Owen, and many men, spoke of disconnects in which both the physicality and validity of their relationship was lost. This disembodied crisis also permeated men’s COVID-19 lives more generally, as Owen disclosed: “I have my anxieties and mental health things … I have my own problems” in conceding that in his partnership, “I was the one who probably needed the most emotional support.” Many men also explained the challenges for virtually reading their partners as well as expressing their own emotions. Oliver, a 32-year-old heterosexual man in a 1.5-year long-distance relationship, spoke to the disconnects invoked by the pandemic restrictions:

I think there’s a consensus out there that communication is one of the most important things between two people in the setting of a relationship … the fact that this was compromised by factors beyond our control was a big part of what happened.

Also implicated in these long-distance relationship break-ups was great uncertainty about when the pandemic restrictions might ease the challenges for trying to be virtually together.

Couples also grew apart under the pressures of living together during the pandemic. The intensity of, and reliance on, the relationship challenged many men, with a lack of activity outside the partnership being a major stressor. Some participants moved in with their partner during the pandemic, oftentimes out of convenience, to be together (rather than alone) in the COVID-19 restrictions. This was the case for Luke, a 28-year-old heterosexual man, who met his partner of 1 year at university just prior to COVID-19. Opting to move into an apartment together when the pandemic hit, Luke recalled that at the time, “I never really had expectations … I’m more of a guy who’s like, ‘Let’s see what happens.’” Unfortunately, disconnects were quickly evident:

We couldn’t really leave our apartment … I think just with that kind of mental attack, “I’m with this person for God knows how long, and there’s only four walls here, I can’t do anything, I just get to talk to this person,” like, it got very challenging because of the COVID pandemic. I think if the pandemic didn’t exist, I think we would still be dating right now.

Luke detailed their mutual withdrawal in the apartment, with silence (rather than arguments) characterizing their standoff, ahead of the relationship officially ending when their lease was up. Reflecting on their partnership, he said, “being together was tough and we couldn’t express that … we both went into depression … we couldn’t control ourselves mentally.” Luke’s vulnerabilities ruptured his claims on idealized masculinities that were characterized by being rational, resilient, in control, and ever-able to protect partners.

Some men had been living with their partner pre-COVID-19, and the end of these longer-term relationships was linked to pandemic-related incompatibilities. Connor, a 27-year-old heterosexual man who had lived with his partner for 4 years, described the negative effects of ever-growing distress in his partnership:

I think over time being forced to live together under those circumstances and pushing trust onto each other … it started highlighting a lot of our insecurities—a lot of my insecurities and a lot of her insecurities as well. It presented more challenges than I was willing to take on. I think it was just added challenges—that sort of outweighed the cons versus the pros in the relationship.

Connor contextualized the added distress in his and his partner’s different interpretations of and compliance with the COVID-19 restrictions. Specifically, Connor’s partner had questioned his actions for putting her at risk after he disclosed having briefly visited a friend’s parents. His commitment to protecting each other from COVID-19 was questioned, along with queries about his need to connect socially outside of their relationship. Increasingly dissatisfied with his partner’s assertions and accusations, Connor listed a myriad of “misalignment perspectives” negatively impacting every aspect of their lives, including disputes arising from both of them needing to work from home. Progressively experienced as demands and ultimatums, Connor moved out, suggesting his partner had become intolerably controlling. While participants’ practices regarding emotions and communication varied (from reliance on partners for emotional supports to mutually concealing or conflicting emotions), Connor, and many participants, spoke to the dislocating forces of the pandemic for *their* intimate partnership.

In sum, couples who lived apart and those who cohabitated reported mental health challenges that affected the quality of their relationships, with the disconnects and burdens outweighing the benefits of being together. Fore-fronted were the divisive forces of COVID-19 for ending the relationships.

### Mentally Trapped, and Failing Fast and Slow

Participants spoke to being mentally trapped in their relationships, and failing both fast and slow. Here, pre-existing as well as emergent mental health challenges recursively flowed to and from participants’ relationships. Consistently linked was a lack of agency to curb the social isolation and its effects. Alex, a 19-year-old bisexual man living separately in a 6-month relationship, explained that being “cut off from [his] regular … support systems, [and] friend systems” put a lot of pressure on his mental health and partnership:

Texting can often be contrived in a way by my brain as, “if somebody isn’t getting back to you, they’ve seen it, and they’re ignoring you, or they’re not, if people aren’t responding to you immediately like they would be if you were in front of them talking to them, there’s something wrong.” Obviously, that’s not the case, and people have their own lives, aren’t always on their phones, but in that period of time [COVID-19 lockdown], I was—suffering from something that would eventually be diagnosed as an anxiety disorder.

Somewhat ironically, Alex’s anxiety was heightened by reaching out to others for the social connection he craved, leaving him mentally trapped in his worries about being too needy and/or ghosted. His partner, also facing mental health challenges, suggested pausing their relationship to “work on herself”—a proposition Alex impulsively reacted to by ending the partnership rather than “taking a break.” Admitting that the break-up worsened his isolation and anxiety, with a year-long distance from the split, Alex offered some clarity about the causative agents: “Our symptoms became so strong and exacerbated by COVID, we were just basically two anxious people trying to console each other about not being anxious.” Challenges for being mentally trapped in distressed states and relationships were evident, and Alex’s abrupt end to the partnership, amid an aftermath of ruminating about the break-up, illustrated how relationships could simultaneously fail both fast and slow.

There were significant challenges in the wake of men’s relationship break-ups, especially when COVID-19 restrictions were still in place. Liam, a 32-year-old heterosexual man in a 3-year relationship with a woman 8 years his junior, suggested that plans for buying a property and moving in together were stalled by COVID-19. The pandemic restrictions left his partner living with her family and focusing on her parents’ financial challenges, to the extent that she ended the relationship with Liam. Explaining the isolating effects of the break-up, Liam said:

You’re talking to someone like every day, and then suddenly they’re just kind of not there anymore. It’s like your whole daily routine is thrown off, and you feel that gap—like something’s kind of missing. So, I think one of the things that would have definitely helped at the time was having someone that I could open up with and talk to. It’s like, when you sit with your own thoughts, you are kind of your own worst enemy at the time. You’re justifying this thing, but it might be reinforcing the wrong kind of situation or scenario that you have in your mind. Having someone to bounce ideas off of and kind of bring you back down to earth, and saying like, “Okay, definitely my mindset was completely off here. Here’s another unique way to look at it.”

Mentally trapped in his afterthoughts, Liam spoke to the absence of in-person supports after the relationship ended. Again, failing fast and slow, he lamented having missed the warning signs, including his partner’s disinterest in buying a house as being reflective of wider divisions in their partnership: “She was just really dismissive of it, like, ‘oh, I’m not putting anything towards this. I’m not paying for any of this. It’s like, that’s kind of your problem to deal with on your own.’” For both Alex and Liam, their post break-up ruminations manifested a sense of deficiency, including failing to see and/or remedy a relationship in decline. That Alex initiated his break-up for fear of being dumped, and Liam lamented the lack of opportunity to save his partnership, underscored their cravings (and shortfalls) for controlling the narrative about and outcome of the relationship. Control, as a masculine ideal, was lost and ultimately undone by their respective partner’s (imagined and real) rejection.

The temporal dimensions for failing fast were also explicitly discussed by some participants. Nathan, a 43-year-old heterosexual man, who met his partner just before COVID-19, explained they wanted to “do this [pandemic] together—not alone” in deciding to “basically become each other’s bubbles” by moving in together. Reflecting on the experience, he suggested:

It kind of, almost quickened the lifecycle of our relationship. That’s kind of how I felt. It was like the pandemic can really just magnify it. In some ways, it probably saved a lot of people years of not asking each other the right questions … For me, the breakdown was … It was just this question that was always pushing, “What are we doing? What are we doing after?” So, maybe that’s why these relationships were hyper-cycles … where a month is a year.

The criticality of the relationship as well as the uncertainty for when and how it might go forward manifested tensions wherein Nathan quickly knew that their partnership had failed. In these cases, there was certainty that the incompatibilities were not entirely about the pandemic but rather COVID-19 bringing the intensity of their discords into focus quicker.

Relatedly featured were divisive outcomes that randomly flowed from the pandemic. Sam, a 50-year-old heterosexual man, had met his ex-partner of 2.5 years at their shared workplace. When the pandemic hit, they both got laid off, but his partner (who had worked at the company for 3 years) was rehired while Sam was fired after having worked more than a decade for the same company. He explained:

They terminated my employment and wanted to pay me the bare minimum … which is 8 weeks severance pay … I had appealed the whole process … I was pissed off at … how they were treating me … Doing all the paper work, had to eventually file with the Court … that was a huge stress for me. And [my ex-partner] was just still going to work every day at this [company] and then I was bad talking them. So it just got more awkward … I didn’t necessarily want to work on it while she was at home cause it just put me in a very nonplussed mood.

Losing his career and paid-work to COVID-19 induced significant hardship, and Sam’s partner’s re-employment deeply contrasted and inflamed his injustices. Herein, his marginality rose, both in the sudden imbalance for what had been a dual-income partnership as well as the institutional disregard and cumbersome legal processes for trying to re-dress that subordinate state. Sam’s mental health challenges grew with his lost autonomy and purpose (both masculine ideals). These deficits were invoked, in large part, by the patriarchal structures Sam had been complicit in sustaining, a betrayal fueling resentment and separate lives in a relationship forever changed and inevitably ending. COVID-19 effects were the nexus of Sam’s challenges—and his relationship failing fast and slow reflected a series of painful unfolding events.

Mentally trapped, and failing fast and slow underscored participants’ lack of control over their circumstances with wide-ranging injuries hastening, but also drawing out men’s distressed and disrupted relationships.

### Introspections and Moving On

While participants experienced mental health challenges with their relationship ending, their introspective learnings mapped across a continuum to bolster their efforts for moving on from the break-ups. Some men were early on in processing their emotions about and experiences of losing their relationship in the pandemic. Benjamin, a 24-year-old heterosexual man who initiated the break-up with his partner of 3 years, recalled the distress he felt leading up to officially opting out of what had become a caregiver role for him working to quell his partner’s mental health struggles:

In the last six months of our relationship … I couldn’t be alone with my thoughts because that was all I was thinking about—breaking up with her … I could barely even listen to music at that point because it wasn’t enough of a distraction.

Reconciling the guilt he harbored about leaving his partner, Benjamin retrospectively summated, “Being brutally honest is the best thing to do, no matter what … the minute you start lying to yourself, it’s game over.” He elaborated that his own mental health was bolstered by the professional care he accessed after the break-up—suggesting the introspections primed by his therapist helped him work through concerns that “I wasted her time” amid his desire and actions for moving on to “explore a new life.” Benjamin understood he could not rush this process work, conceding that he needed to keep working with professional help to fully achieve his goal of building a healthier relationship in the future.

Many men reached out to friends and family for support to process and understand their own shortfalls in the relationship and its end. Jacob, a 32-year-old heterosexual man, was adamant that poor communication led to the breakdown of his 3-month relationship. Littered with conflict, he suggested that the miscommunications with his partner cyclically flowed to and from their unending arguments:

I needed a day after [an] argument to just step away. And she took that as a big sign of me basically walking away from the relationship … it got to a point where it felt like neither of us were being heard, validated or understood.

Recognizing “a tremendous amount of grief,” Jacob intentionally opened up to “a lot of girl friends and some guy friends” to express and guide his introspections for all that had happened in and to end the relationship. Again, the focus was on what Jacob might have done better rather than trying to assign blame or unravel his partner’s behaviors. Consistent in Benjamin’s and Jacob’s introspections were deliberate engagements with external help for doing the work of understanding and more wisely moving on from the relationship. Evident in these accounts was participants’ strength-based positioning of their self-work in ways that marked alignments to masculine ideals for being authentic and accountable for their own behaviors.

Participants beginning new intimate partner relationships also featured as a means to moving on from their COVID-19 break-ups. In these contexts, men suggested they were making corrections based on what they had learnt from their break-ups. Noah, a 23-year-old heterosexual man in a new relationship, emphasized the importance of compatibility and really knowing your partner in deciding to have an exclusive partnership. Recalling how he had unwisely ignored “red flags” including his ex-partner’s smoking and their incompatible life goals, Noah was purposefully taking things slowly in his current relationship. This included explicitly discussing values and visions for achieving what was important to them as individuals and as a couple:

We have very similar places in our life … I made sure that the person that I’m currently seeing is someone that will have the same schedule, we align with our political views and personal goals where we can uplift each other.

Detailing a “thorough screening” process, Noah suggested he and his new partner were ideally matched in their compatibility and joint commitment to communicating to smooth the inevitable shifts and transitions in their relationship. Driving these adjustments for many men were the mental health challenges that had flared in their distressed COVID-19 relationships and break-ups. Being more cautionary and conservative about forging intimate partner relationships for self-protections were evident. For example, Nick, a 25-year-old heterosexual man, described experiencing emotional abuse toward the end of his 7-year relationship:

There were a few things that were said that were very hurtful … during the break-up, just like “Oh, I don’t find you attractive” … there was a lot of mean stuff … she also said something about me being too short.

Nick elaborated this abuse as a pattern in the relationship, lamenting that COVID-19 and its restrictions had led him to “accept a lot more disrespect and other things that [he] shouldn’t have.” Summating that he felt like he “was just being an inconvenience” to his partner, a relational pang that left him trying to “withhold anger”, Nick recognized the dire effects on his mental health: “I would be really sad and barely able to function…and work became difficult.” Nick’s introspections focused on his complicity in the distressed relationship as a means to moving on from and never again accepting such abuse in a partnership. He tallied, “It taught me about healthier boundaries, and things that I should and shouldn’t accept” and to have “more confidence in myself and in my own value.” Nick’s and many participants’ introspections were dedicated to self-growth, and while moving on typically involved a recovery component, longer-term goals and gains were pursued through men’s post-break-up work. As Jackson, a 21-year-old heterosexual man, speaking about his 2-year relationship asserted: “You have to put yourself first. You can’t love someone if you don’t love yourself.” He went on to say that honestly expressing what was felt was key to building trust with intimate partners:

My new partner and I were having a conversation recently, and she doesn’t think her love is the same as it was once a while ago. I don’t think mine is either, I don’t think I love the same way.

Jackson’s candid account indicated his recognition that relationships shift with time and context, and rather than fatalism about an impending end to the partnership, he viewed discussing differences and/or conflicts and expressing *their* truths as nourishing. Here, the introspections about what Jackson did not or could not do in his failed COVID-19 partnership drove his work for moving on to more authentically and competently showing up in his new relationship. Men’s most often told vulnerability resilience narrative was echoed, wherein Jackson’s struggles were background to his decisive actions for forthrightly articulating, accepting, and adapting to the shifts in his partnership.

Participants’ introspections and moving on illuminated strength-based masculinities wherein seeking help and self-work focused on deconstructing their challenges to develop healthier relationship values and visions, and in some instances explicitly working those revelations into new partnerships.

## Discussion

The current study findings provide important insights to men’s intimate partner relationship break-ups during COVID-19. Participant experiences and perspectives have structural and behavioral implications amid providing direction for developing gender-responsive mental health promotion programs that are explicitly focused on men’s relationships. In what follows, the three thematic findings are discussed in making recommendations for upstream mental health promotion services for men.

In theme one, *Virtually Together and Growing Apart*, the impact of COVID-19 and its restrictions were highlighted as disrupting both virtual and live-in relationships. Underscoring the distance and absence of receptive touch for forging emotional intimacies, the limits of virtually connecting amid great uncertainty for how long those constraints might last, contributed greatly to men’s distressed and disrupted relationships. There were also unprecedented pressures on the domestic sphere whereby many live-in partners had to work from home, and/or exclusively rely on each other to satiate their social connection needs—entwinements that Pietromonaco and Overall (2021) have previously reported as troubling many relationships in the pandemic. The current study findings also help to contextualize Statistics Canada (2021) reports that during the pandemic, intimate partners experienced increased stress wherein wide-ranging relationships battled to overcome pandemic induced challenges. This is not to deny the potential for pre-existing relationship issues as inevitably ending partnerships during but being independent of the pandemic, nor is it to assign COVID-19 as the sole causative agent in the participants break-ups. Rather, what is helpful to moving forward is to understand the pandemic as an ongoing transition, similar to the inevitable (and sometimes unforeseen) changes that routinely challenge intimate partnerships (e.g., illness, debility, bereavement, parenting, and retirement). That said, key to men’s relationships and mental health services are trauma-informed care approaches that focus on deconstructing these injurious events as the foundation for working through challenging life events and transitions.

The findings related to *Mentally Trapped, and Failing Fast and Slow* confirm and build upon previous research describing the entwinements of men’s mental illness with distressed and disrupted intimate partner relationships (Evans et al., 2016; Oliffe, Kelly, Gonzalez Montaner, Seidler, Ogrodniczuk, et al., 2022; Scourfield & Evans, 2015). Parsed also are previous COVID-19 study findings connecting low relationship quality and increased risk for depressive symptoms (Charvat et al., 2023; Zoppolat et al., 2022), and the recognition of loneliness, longing, and sadness as moderated by long-term forced separation (Samokhvalova et al., 2022). The current study findings add to these insights by revealing pre-existing mental illness in one and/or both partners as deeply implicated in relationship breakdowns during COVID-19. Also evident were unique challenges for men accessing professional mental health care and peer supports during the pandemic. That relationship break-ups and mental illness are among the strongest predictors of men’s suicidality (Richardson et al., 2021) underscores the critical need for community-based mental health promotion programs to help [re]address the traumatic aftermaths of distressed/disrupted partnerships in COVID-19. Recent reports of a loneliness and isolation epidemic (Office of the Surgeon General [OSG], 2023) along with public health’s pandemic induced bankruptcy (Oliffe et al., 2024) have lobbied community care and peer mutual help (Sharp et al., 2024). Here, the scaling and sustaining of existing upstream programs such as Man Cave, Men Building Intimate Partner Relationships, and In Good Company are critically important to engaging and equipping men with strategies for ensuring their mental health. The increase in men’s use of telehealth also suggests that community-based gender-responsive mental health promotion programs might effectively integrate virtual and in-person services (Ziesing et al., 2022).

The third theme, *Introspections and Moving On*, echoes previous research suggesting men’s help-seeking is inclusive of friends, family, and/or professional mental health care to cope with and/or garner growth through relationship break-ups (Entwistle et al., 2021; Fletcher & StGeorge, 2010; Oliffe, Kelly, Gonzalez Montaner, Seidler, Kealy, et al., 2022). Adding to these insights, the current study findings reveal many men as seeking assistance (and perhaps permission) for doing introspective work for self-growth. As Oliffe (2023) has detailed, vulnerabilities and resilience are common masculinity storylines, and the current study underscores the value of men’s strength-based work for accountably working through their challenges to effectively move on. Mental health promotion programs might usefully include permission and affirmation to sustain men’s introspections as foundational to maintaining healthy intimate partner relationships. Nestling such work as recovery and growth is vital for men to avoid repeating negative patterns in future relationships. Recent work in men’s gender equity and gender equality also seems especially important for relationship programs and services that are dedicated to legitimizing contemporary masculinities and intimate partner relationships (see Oliffe et al., 2023).

In concluding the current study findings discussion, beyond men’s partnerships and their individual and relational behaviors, it is crucial to examine and authentically account for the structural determinants of men’s mental health, as Heller et al. (2024) have recently and eloquently argued. Said simply, while the inconsistent COVID-19 messaging, governance, and policing that unraveled worldwide cannot be annulled, those structure shortfalls highlight how stripping people’s agency and feeding uncertainty can wield profoundly divisive relational effects. The extent of the pandemics structural harms will of course never be fully known (or acknowledged), but the high and rising demand for mental health care and the long waitlists for family law courts to process COVID-19’s disrupted relationships underscore the influence (and obligations) of health policy and legislation. Within this context, it is fair to say that masculinities and men’s mental health promotion research has long grappled with agency and structure, exhibiting a propensity for lifestyle drift wherein the gendered dimensions of men’s behaviors are studied and explained amid the offering of tailored interventions designed to shift *their* maladaptive practices (Oliffe et al., 2024). COVID-19 beckons actions for integrating and addressing the structural determinants of men’s ill-health—with the goal of nimbly adjusting policy and legislation as well as building and formally evaluating gender-responsive mental health services.

Study limitations include the cross-sectional design and reliance on men’s perspectives, which shorts what can be known about changes over time and the relational work leading to the partnership break-ups. That the sample was small, and participants were Canadian-based, with most of them <30 years old also limits the reach of the findings. Future work might helpfully address these limits with longitudinal couple-dyad studies comparing age and locale specific sub-groups to test and advance the current study findings.

## Conclusion

While we cannot change COVID-19’s past, there are clearly moral and ethical imperatives driving the need to make good on promoting men’s mental health in the aftermath of the pandemic. There are of course many COVID-19 casualties in terms of what was socially and emotionally lost (agency and intimacy), and the long reach of those injuries should be both anticipated and effectively treated. While population and public health chronicles the epidemiology of men’s mental illness problems, politics and policy needs to hear from and respond to the individuals who were impacted. To this end, the current study offers highly relevant relationship break-up narratives as a means to guiding men’s gender-responsive mental health promotion services and programs.
